# Effect of Elevated Temperature on Rhyolitic Rocks’ Properties

**DOI:** 10.3390/ma15093204

**Published:** 2022-04-29

**Authors:** Haitham M. Ahmed, Mohammed A. Hefni, Hussin A. M. Ahmed, Sefiu O. Adewuyi, Ferri Hassani, Agus P. Sasmito, Hussein A. Saleem, Essam B. Moustafa, Gamal S. A. Hassan

**Affiliations:** 1 Mining Engineering Department, King Abdulaziz University, Jeddah 21589, Saudi Arabia; mhefni@kau.edu.sa (M.A.H.); haahmed@kau.edu.sa (H.A.M.A.); sadewuyi@stu.kau.edu.sa (S.O.A.); hasmohamad@kau.edu.sa (H.A.S.); gshassan@kau.edu.sa (G.S.A.H.); 2 Department of Mining and Materials Engineering, McGill University, Montreal, QC H3A2A7, Canada; ferri.hassani@mcgill.ca (F.H.); agus.sasmito@mcgill.ca (A.P.S.); 3 Mechanical Engineering Department, King Abdulaziz University, Jeddah 21589, Saudi Arabia; abmostafa@kau.edu.sa; 4 Department of Mining and Metallurgical Engineering, Assiut University, Assiut 71515, Egypt

**Keywords:** rhyolitic rocks, heat treatment, microstructure, rock physical and mechanical behavior, failure modes

## Abstract

The effect of high temperatures on rock’s thermophysical and mechanical properties is critical to the design of underground geotechnical applications. The current work investigates the impact of temperature on rhyolitic turf rock’s physical and mechanical properties. Intact cylindrical core rock samples were heated to different temperatures (200, 400, 600, and 800 °C). The uniaxial compressive strength (UCS) and elastic modulus of unheated and heated samples were determined as important mechanical properties. In addition, the effect of temperature on the physical properties of rhyolite rock (density, color, and absorption) was investigated in conjunction with its microstructural properties. The hardening of the rhyolitic rock samples was observed below 600 °C, at which point the UCS and elastic modulus decreased to 78.0% and 75.9%, respectively, at 800 °C. The results also show that heating does not significantly affect the density and volume of permeable pore space, but a color change can be observed at 400 °C and above. A microscopic analysis shows the change in microstructural properties of rhyolite rock after heating to 600 °C. Furthermore, the SEM observations of heated materials show structural particle displacements and microcracking, leading to apparent surface cracks.

## 1. Introduction

Rocks are made of different geological formations that determine their thermophysical and mechanical properties that may be affected by high temperatures [1,2]. With the increasing complex geological conditions of rock formation in various underground projects worldwide, the effects of temperature on rock’s physical and mechanical properties are of great importance [1]. This is due to its wide range of applications, including archaeology [3,4], nuclear energy storage and waste disposal [5,6], earth structure and petrolitic exploitation [7], earthquake mechanisms, geothermal power generation [8,9], building and road construction [10,11], hydrothermal system [12], thermal energy storage (TES) [13,14,15,16], underground coal gasification [17], underground rock tunnels [18,19] and deep mining engineering [20].

For information on changes in the physical properties (including P-wave velocity (V_p_), density (D), porosity, and color) of rocks due to the temperature increase, numerous studies can be found in the literature. Sun et al. [21] studied the effect of temperature on the P-wave velocity and microstructure of sandstone samples. Their results showed that the sandstone samples’ average P-wave velocity change rate fluctuated between 22 and 450 °C and later increased up to 68.81% at 900 °C. A comparison of Scanning Electron Microscope (SEM) images among the samples at room temperature and those heated to different temperatures showed that the cementation property of the particles became poor, and many pores were developed on the heated samples [21]. In a similar study using granite, findings indicated that the P-wave velocity of the samples decreased throughout the tested temperatures up to 900 °C [22]. The fastest decrease in P-wave velocity was 52.7% at a temperature range of 400–600 °C [22]. This may be attributed to a change in the quartz phase from α to β around 573 °C [20]. Microstructurally, SEM photographs of the studied quartz samples showed that different degrees of cracks were developed after heating [22]. Liu and Xu compared the effect of temperatures on P-wave velocities of sandstone and granite. Their results revealed that the rate decrease in P-wave velocity in granite was far greater than sandstone under the same heating conditions [23]. As for the density and color, the studies have shown that they change by increasing or decreasing as a result of heating, as it appeared in the samples of granite rocks, sandstone, microschist, and limestone. The studies have further linked the impact of heating on microvoids to the displacement of impurities and have highlighted the presence of iron in the rocks as a possible reason for the change in color of rocks after heating [20,24]. Additionally, the evaporation of weakly bounded water, and strongly bounded water in the rock’s mineral composition occurs around 150 and 200 °C, respectively, which has been linked to rock weight loss and a subsequent decrease in density [25]. A change in the mineral phases of rock after heating has also been related to a decrease in density [25,26]. The porosity increases with increasing temperature due to the grains’ expansion and the formation of microcracks after heating [27,28]. However, the degree of increase in porosity varies from different types of rocks [27,28]. 

Several studies have been conducted indicating that the mechanical properties (such as uniaxial compressive strength (UCS), modulus of elasticity (E_A_), and tensile strength) of rocks can change (increase, decrease, or show a mixed change) after heating to a certain temperature (Table 1). The post-heating change in mechanical properties of rocks depends on factors including mineralogy/rock type, heating rate, cooling rate/medium, heat-soaking time, and target temperature. Irrespective of any of these factors, researchers have pointed out that temperature may significantly affect rock’s mechanical properties [29]. Many of the previous studies have been carried out majorly on sandstones and granites [20,30,31]. It was found that as the target temperature increased, the UCS, E_A_, and tensile strength of granite decreased [32]. Shao et al. reported similar results that UCS and E_A_ of granite decreased with increasing target temperature up to 1000 °C [33]. Some researchers found that below 400 °C, granite showed a slight change in UCS, while the UCS drastically dropped above this temperature. Therefore, 400 °C was regarded as the threshold temperature for the investigated granite [34,35,36]. Establishing a threshold temperature and the thermal behavior of every rock type is one of the scientific needs and still receiving the attention of researchers [37]. The threshold temperature of carbonate rocks, sandstones, mudstones, and shales are 300–400 °C, 300–500 °C, 500–600 °C, and 600–700 °C, respectively [38]. Further information as per threshold temperatures of some rocks can be found in the literature [39]. As presented in Table 1, it can be noticed that the heating of sandstones caused a series of complex thermal behaviors; it may increase the strength of the rocks or result in the degradation of the rocks. Studies regarding the effect of temperature on the rhyolitic rock are scarce in the literature despite being widespread in the metamorphic region worldwide and specifically in the Najran area of Saudi Arabia. This rock type usually occurs due to metamorphic deformation and alterations that may lead to the recrystallization of grains and variation in structural properties [20]. When such rocks are subjected to heat, their heating behavior and the consequent effect of heating on their physical and mechanical properties may vary due to their structural variation and may undermine the structural integrity of the rock mass in engineering structures [39]. Establishing the threshold temperature of rhyolitic tuff rocks and understanding thermal effect on their mechanical and physical properties are important for structural engineering planning purposes. 

Despite the widespread use of rhyolitic tuff rocks and their application in civil engineering and underground constructions, there is a lack of literature on the effects of high temperatures on this type of rock. Therefore, the current investigation aimed to study the effect of temperature on rhyolitic tuff rocks, focusing on the changes in their mechanical (uniaxial compressive strength and elastic modulus), physical, and microstructural properties rocks. Moreover, the prediction of the fracture mechanism of the rock from their anisotropic nature has been studied; hence, it may create severe structural engineering. 

## 2. Materials and Methods

### 2.1. Sample Preparations and Characterizations

**Figure 1:**
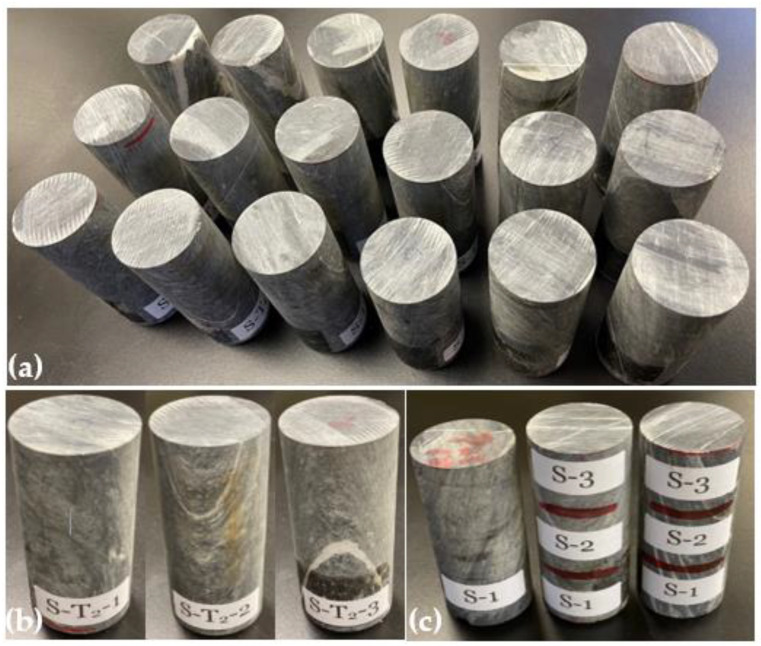
Prepared drill-core specimens: (**a**) eighteen prepared specimens; (**b**) a set of specimens to illustrate selection criteria for experimental work, to be tested at temperature 2, i.e., 400 °C; (**c**) specimen to the left; for physical properties measurement, middle specimen; for chemical/mineralogical/morphological analysis, and specimen to the right; for petrographic analysis. S-1, S-2, and S-3 represent a specimen at room temperature (22 °C), a specimen for the heating experiment at 600 °C, and a specimen for the heating experiment at 800 °C, respectively.

In this study, eighteen rock specimens (Figure 1a) were prepared using diamond drill-core rock samples obtained at the Najran area of Saudi Arabia. The intact drill-core samples were cut (using PT 100 GTCS Testing Systems, Tempe, AZ, USA) and surface-polished (using KGS618, Tustin, CA, USA) to 0.04 surface tolerance according to the ASTM standard D4543 [44]. The specimens were then dried at 105 °C for 24 h using a laboratory dryer (Binder Inc., Bohemia, NY, USA). Fifteen prepared specimens were labeled according to the test temperatures; T_0_, T_1_, T_2_, T_3_, and T_4_ are 22 (room), 200, 400, 500, 600, and 800 °C, respectively. Meanwhile, three samples were systematically selected for each target temperature based on their macroscopic differences in mineralogy, bedding, and foliation plane; the first sample had no visible quartz-carbonate vein, the second sample had a visible quartz-carbonate foliation plane, and the third one had a major quartz-carbonate vein, bedding, and foliation plane (Figure 1b), for quality assurance and quality control purposes and being able to analyze the behavior under these different foliation styles. Therefore, the three samples at 22 °C were, respectively, labeled as S–T_0_–1, S–T_0_–2, and S–T_0_–3 (where S represents a sample, T_0_ means room temperature, and 1–3 are the sample numbers). 

A dedicated specimen was selected for chemical, mineralogical and morphological analyses (middle specimen in Figure 1c). This specimen was cut into three pieces ((Figure 1c; S-1–S-3)), representing different testing target temperatures (22 (S-1), 600 (S-2), and 800 °C (S-3)). The specimens at these testing temperatures were separately comminuted using a laboratory jaw crusher (BB300 Mangan Retsch, Haan, Germany), a roll crusher (Sew-Eurodrive GmbH & Co KG, Bruchsal, Germany), and a ball mill (Sew-Eurodrive GmbH & Co KG, Bruchsal, Germany), till 100% particles passed an 80 µm sieve. Higher temperatures below 600 °C were not selected for the aforementioned analyses because the observation of the specimens shows little or no change in physical appearance. This may be related to the thermal stability of the specimens as reported for the selected rock samples in the same region [20,45]. The chemical (elemental) analysis was performed using a Field Emission Scanning Electron Microscope-coupled with Energy Dispersive X-ray (FESEM-EDX: FESEM JSM-7600F, JEOL Ltd., Tokyo, Japan; EDX spectrometer: Oxford Instruments, Oxford, UK) [46,47]. Meanwhile, the SEM images of the studied specimens were captured at different magnifications for morphology analysis purposes. Furthermore, mineral phases in the specimens were examined using the X-ray Diffraction (XRD) method. The XRD spectra of the specimens were taken at a scattering angle 2θ between 10 and 80°, with a step of 0.05° in a continuous mode operation, using an X-ray diffractometer with a Cu kα radiation source (Regaku, Ultima 1V, Tokyo, Japan) [46]. The obtained XRD data were processed using “Match!” Software (Version 3.12, Crystal Impact GbR, Bonn, Germany) matches the spectra with the standard ones. The quantitative analysis of the XRD spectra was performed using the relative intensity ratio (RIR) embedded in the “Match!” Software.

Similar to a specimen prepared for chemical, mineralogical, and morphological analyses, a cylindrical drill-core specimen (specimen to the right in Figure 1c) having required characteristics considered for the specimen categorization was cut into three (Figure 1c; S-1–S-3); each representing target temperatures 22, 600, and 800 °C for petrographic analysis. Each specimen was physically examined by stereomicroscope (Leica S9i, Leica Microsystems Inc, Buffalo Grove, IL, USA) and a standard petrographic thin section was prepared along the representative area of the specimen. The polarizing microscope (Olympus BX53M, Tokyo, Japan) was used for specimens’ transmitted light observations. At the same time, the digital camera Olympus SC180 was employed to capture the photomicrographs of a different area of the thin-section specimens. For more specific details of the effect of temperature on the studied samples, SEM photomicrographs of powder samples at 22, 600, and 800 °C were captured by FESEM (JSM-7600F, JEOL Ltd., Tokyo, Japan). 

The physical properties analysis of the studied rock samples was performed using a separate drill-core specimen (Figure 1c; specimen to the left). To obtain the density, the dimensions of the specimen, including height and diameter, were measured and its mass was determined using a digital laboratory balance (Mettler PE 3600, Mettler Toledo, Columbus, OH, USA). The volume and density of the specimen were then calculated. The absorption and void in the specimen were determined using the established ASTM C 642–97 standards [48]. The oven-dry mass, saturated mass after immersion in water for 48 h, saturated mass after boiling for 5 h, and immersed apparent mass were all determined as described in the ASTM standard C 642–97 [48]. This procedure was repeated for a separate specimen heated to 600 °C to observe the effect of temperature on the void space in the specimen. Additionally, as part of the physical properties of the studied rock samples, the P-wave velocities of all specimens at room temperature were measured as described in the ASTM D2845-08 protocol [49].

### 2.2. Heating Process

**Figure 2:**
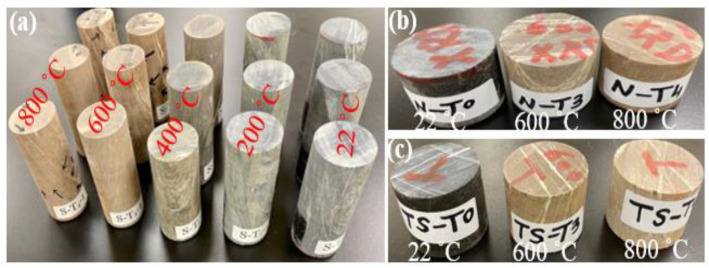
Specimens before and after heating: (**a**) specimens for experimental work; (**b**) specimens for chemical/mineralogical/morphological analyses; (**c**) specimens for petrographic (thin section) analysis.

In this study, three specimens were heated at once at a heating rate of 2 °C/min to the target temperature, using a laboratory furnace (CHM-60H, Jim-bomb Enterprise Co., Ltd., Taiwan). This low heating rate was employed to ensure that the specimen is suitable for the UCS test; it does not break into pieces during the heating process [41]. After reaching the target temperature, the specimens were soaked at that temperature for 2 h to ensure that heat penetrated to the specimens’ matrices before they were naturally cooled inside the furnace to avoid thermal shock [28,41]. The different sets of specimens at all tested temperatures 200, 400, 600, and 800 °C, in addition to the room temperature set, are shown in Figure 2.

### 2.3. Mechanical Properties Experiment

**Figure 3:**
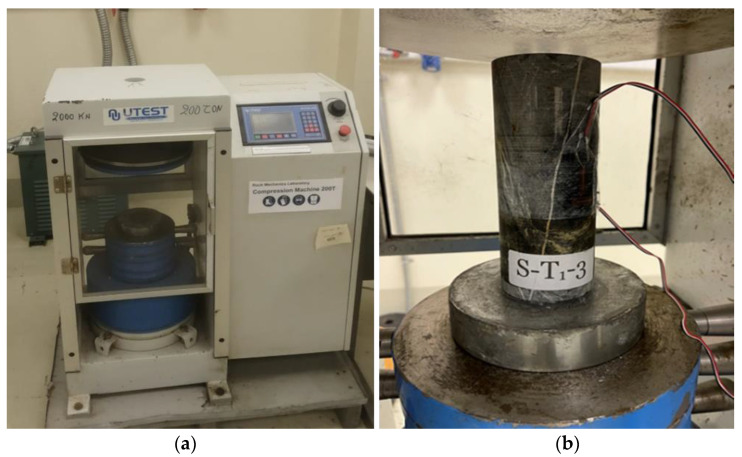
(**a**) Compression machine (Press BC 100, 2000 kN); (**b**) sample S-T_1_-3 during testing in a compression machine.

UCS tests were conducted on the prepared cylindrical specimens using a compression machine with a maximum axial load of 2000 kN (Figure 3, Press BC 100, UTEST, Framingham, MA, USA) at a uniaxial compression rate within 0.5–1.0 MPa/s till the specimen’s ultimate load is reached, according to the ASTM standard D7012-14 [50]. Before the test was conducted, a strain gauge was pasted on each specimen to record the deformation in the axial direction during compression to determine the modulus of elasticity (Ea) for each specimen. As earlier mentioned, three specimens were selected for different target temperatures 22, 200, 400, 600, and 800 °C. The UCS of each specimen at the same temperature category was separately established, and an average of the three specimens in that group was calculated. Additionally, the elastic modulus of the three specimens was separately calculated, and an average elastic modulus was determined. These procedures were repeated for other categories of target temperatures specimens.

## 3. Results and Discussions

### 3.1. Elemental Analysis

Table 2 presents the results of the elemental analyses of the studied samples ST_0_, ST_3_, and ST_4_ 22 °C (room temperature), 600 and 800 °C, respectively. Findings show that the three samples contain O and Si as the major elements, with others at various proportions (Table 2). It can be noted that the major difference in the samples is Ti and Cu; the sample ST_3_ contains Ti as a trace element, while this element cannot be established in the other studied two samples. Additionally, the sample ST_4_ has traces of Cu; meanwhile, this element is not present in the samples ST_0_ and ST_3_. This variation may be related to the heterogeneous nature of metamorphic rocks.

### 3.2. Thin-Section Analysis

**Figure 4:**
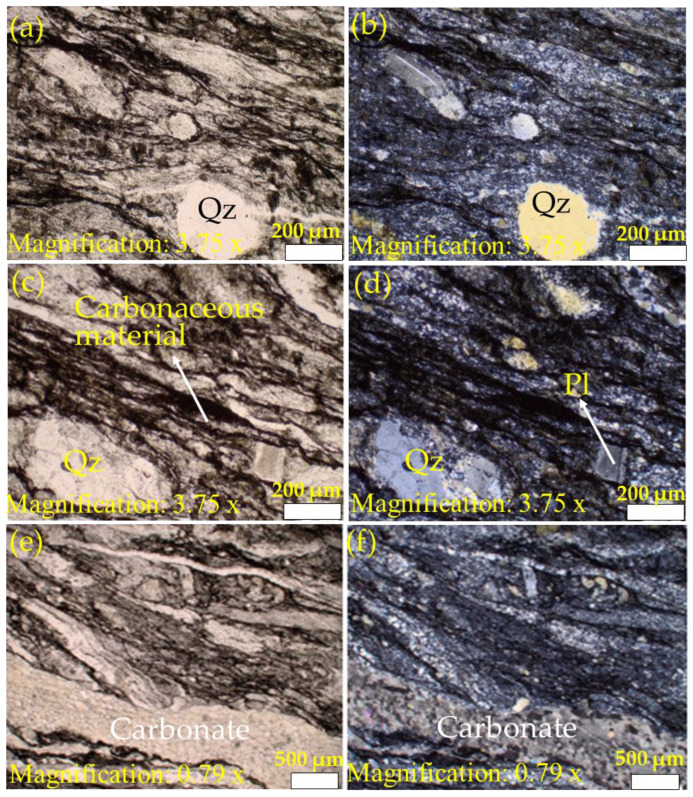
Petrographic analysis of the studied samples at room temperature, 22 °C (Qz and Pl represent quartz and plagioclase, respectively). Images (**a**,**c**,**e**) were taken using plane-polarized light, while (**b**,**d**,**f**) were captured using cross-polarized light. Image (**a**) (PPL) and image (**b**) (CPL) display flaser texture in reworked crystal-rich rhyolitic tuff marked by cataclasis, granulation, shearing, and foliation. Images (**c**,**d**) are close views of images (**a**,**b**) displaying blastoporphyritic texture. Note quartz and plagioclase phenoclasts are set in a recrystallized fine-grained felsic matrix. Images (**e**,**f**) display partial to intense granulation and recrystallization of quartz (Qz) and plagioclase (Pl). Additionally, note dark lamellae of carbonaceous material that follows foliation planes and commonly wrap around phenoclasts. Images (**g**,**h**) show intense shearing and crushing of groundmass and stretching and elongation of phenoclasts parallel to the deformation plane. Additionally, note fractures with quartz and carbonate fillings. PPL: plane-polarized light; CPL: cross-polarized light.

**Figure 5:**
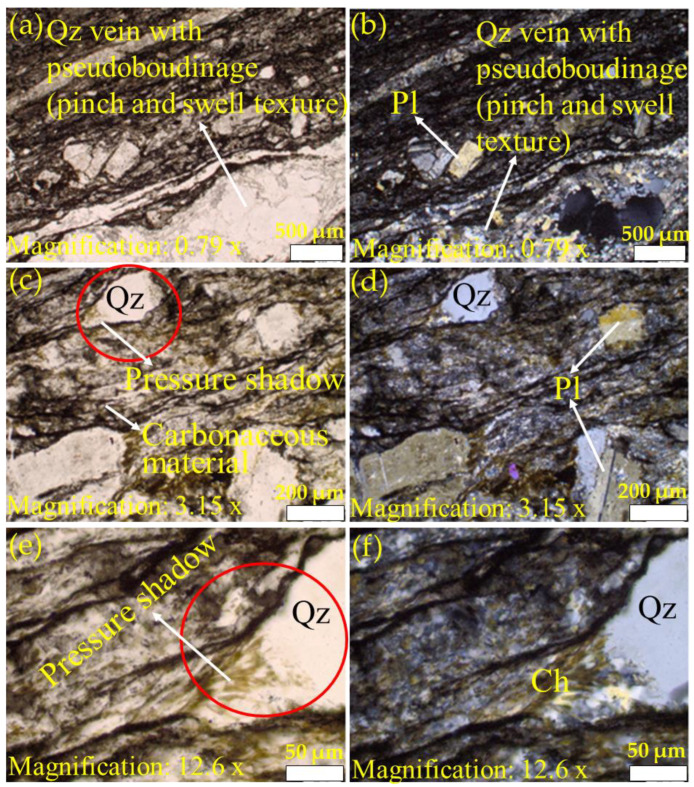
Petrographic analysis of the studied samples at 600 °C (Qz, Pl, and Ch represent quartz, plagioclase, and chlorite, respectively). Images (**a**,**c**,**e**) were taken using plane-polarized light while (**b**,**d**,**f**) were captured using cross-polarized light. Images (**a**–**f**) were taken at different magnifications to reveal rock texture, mineral composition, and alterations in meta-reworked crystal-rich felsic tuff. Images (**a**,**b**) illustrate an excellent example of boudinage where quartz-carbonate veins parallel to shear openings display distinct pinch and swell texture of boudinage. Additionally, note suboriented blastophyres of plagioclase and quartz set in highly sheared and foliated rock matrix in the image (**a**,**b**), while images (**c**,**d**) display unequigranular blastoporphyritic texture. Note highly sheared and foliated felsic rock matrix carrying blastoporphyrites of plagioclase and quartzthat display rotation fabric and the presence of pressure shadows which are marked by granulation and the presence of tiny biotite (partially chloritized) flakes. Images (**e**,**f**) are higher magnification of the red circle area of the image (**c**) to reveal more distinctly pressure shadows. Note that tail-shaped quartz pressure shadows display granulation of quartz blastoporphyrite and the presence of tiny greenish biotite flakes.

**Figure 6:**
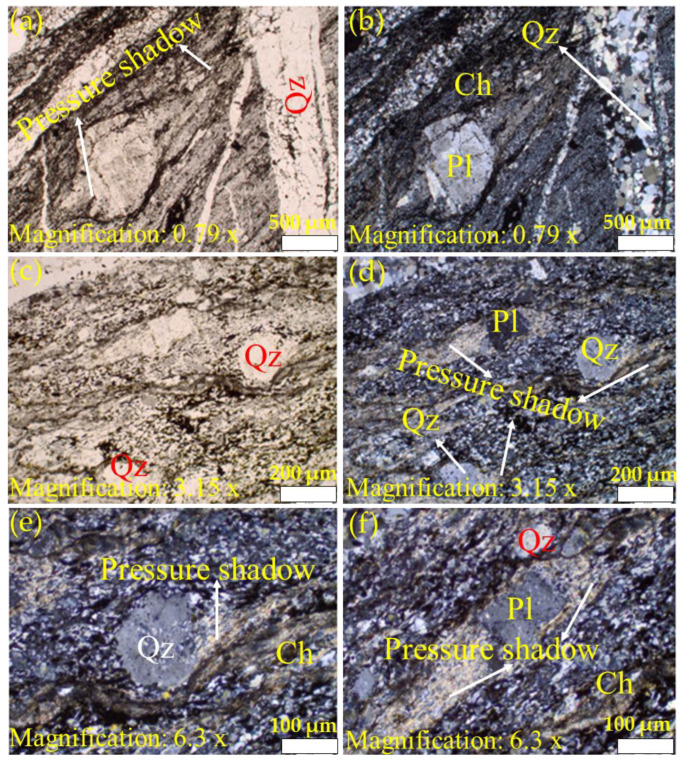
Images (**a**–**f**) were captured at different magnifications to reveal rock texture, mineral composition, and alterations in the meta-reworked crystal-rich felsic tuff. Images (**a**,**b**) display fine-grained unequigranular blastoporphyritic texture. Notice plagioclase and quartz (not visible in this image) blastophyres are set in a highly sheared and foliated microcrystalline felsic rock matrix. Images (**c**,**d**) illustrate that the rock is very highly deformed, mylonitized, and sheared. This is well demonstrated by rotation and the presence of pressure shadows in blastoporphyrites of plagioclase and quartz. Pressure shadows are marked by granulation of blastophyres and the presence of phyllosilicates (biotite/chlorite/sericite). Image (**e**) displays quartz blastoporphyrite granulation and pressure shadows occupied by phyllosilicates. Image (**f**) further illustrates distinct pressure shadows in plagioclase blastophyre, which is occupied by microcrystalline phyllosilicates. PPL: plane-polarized light; CPL: cross-polarized light.

Figure 4, Figure 5 and Figure 6 present the petrographic photomicrographs of meta-reworked crystal-rich rhyolitic tuff, a carbonaceous meta-volcaniclastic rock of felsic composition. Figure 4 presents images obtained from the sample at room temperature (22 °C), while Figure 5 and Figure 6 represent photomicrographs of the samples after heating at 600 and 800 °C, respectively. Petrographic microphotographs were taken at various magnifications to reveal the rock texture, alteration, and mineral composition of the studied meta-reworked crystalline-rich felsic tuff.

#### 3.2.1. Texture and Alteration

Due to intense metamorphic deformation, the primary rock texture was over-printed and altered, which caused granulation (recrystallization), cataclasis, mylonitization, shearing, and foliation subaugen-shaped blastophenoclasts show alignment parallel to the deformation plane (Figure 4, Figure 5 and Figure 6). The studied rock now displays unequigranular, blastoporphyritic, and flaser-fine textures. Late quartz-carbonate veins parallel to shear planes display distinct pinch and swell boudinaged microstructure (Figure 5a,b). However, the presence of blastophyres comprised of plagioclase and quartz are noticed in finely sheared and foliated felsic rock matrix (Figure 6a–f). Subaugen fabric and the local presence of pressure shadows at both ends of plagioclase and quartz blastophyres are apparent (Figure 6a–f).

#### 3.2.2. Mineral Composition

The primary rock composition was completely modified by intense metamorphic deformation and alterations. The rock can be distinguished in (1) groundmass and (2) phenoclasts. The felsic groundmass is recrystallized, very fine-grained and shows foliated fabric. Foliations are marked by the orientation of microcrystalline flaky phyllosilicates (chlorite ± sericite) and microcrystalline dark carbonaceous material and blastophenoclasts of plagioclase and quartz (Figure 4, Figure 5 and Figure 6) that are often partially to completely recrystallized and aligned parallel to the foliation plane or primary depositional plane. Plagioclase blastophenoclasts are platy to tabular in form and range in sizes from less than 0.5 mm to over 0.5 mm in length and display partial to intense recrystallization (Figure 4d,f). Meanwhile, quartz phenoclasts are subrounded in form and range in sizes from 0.2 mm to 1 mm in length (Figure 4d,f). Phyllosilicates (chlorite ± sericite) are microcrystalline, flaky inhabit, and pale-greenish color (Figure 4d,f). The orientation of flaky phyllosilicates grains marks the deformation plane. Phyllosilicates commonly replace and form fine-grained intergrowth over rock-forming minerals. In some places, pressure shadows can be noted at both ends of plagioclase blastophyre and quartz (Figure 5c,e and Figure 6a,d,f). Phyllosilicates (biotite ± chlorite) in Figure 5 are platy in grain morphology and pale-greenish to brownish. The alignment of flaky biotite grains defines the foliation plane. Similar to the micrographs of the unheated sample (Figure 4d,f), biotite commonly replaces and form fine-grained intergrowth over rock-forming minerals. The dark carbonaceous material is amorphous and occurs in thin lamellae, aligned parallel to the foliation plane and wrapping around blastophenoclasts of plagioclase and quartz (Figure 4, Figure 5 and Figure 6).

The only difference noted among the three samples (22, 600, and 800 °C) examined is the change in color of phyllosilicates (sericite/chlorite/biotite). In the sample at room temperature (T-S-T_0_), phyllosilicates are colorless to pale green. In contrast, in the sample heated to 600 °C (T-S-T_3_), they are distinctly brownish-green, and in the one at 800 °C (T-S-T_4_), phyllosilicates are dark. It is not clear whether the change in phyllosilicates color is due to heating or primarily of that color. 

### 3.3. SEM Analysis

**Figure 7:**
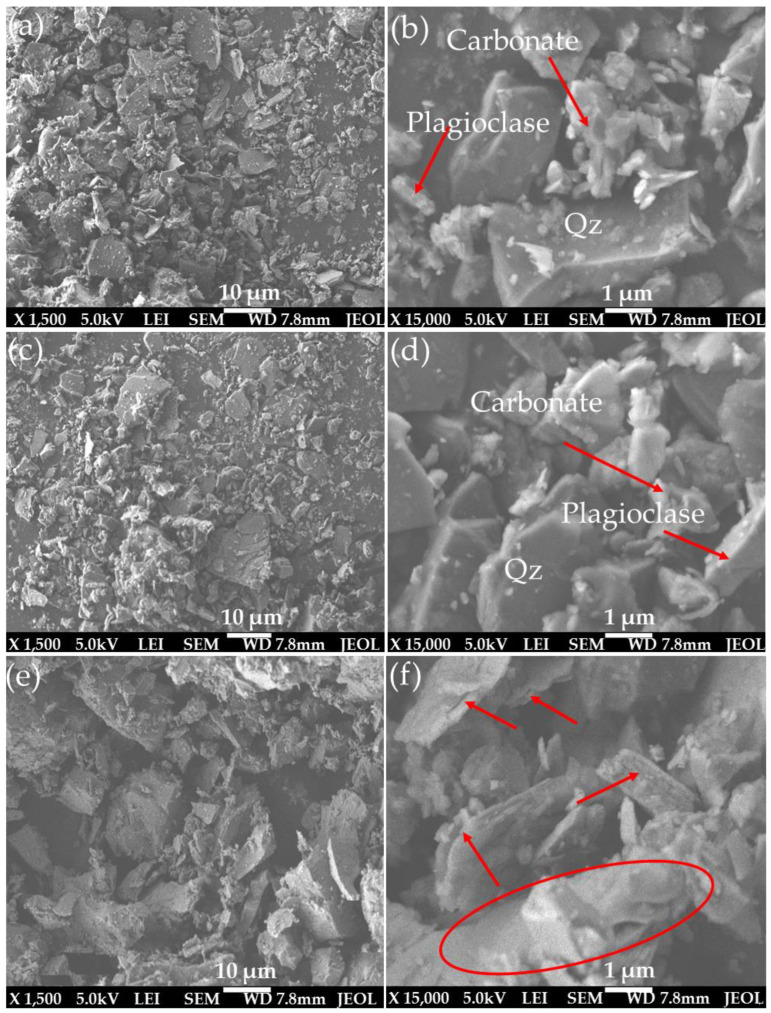
SEM photographs of the studied samples ((**a**,**b**): sample at 22 °C; (**c**,**d**): sample at 600 °C; (**e**,**f**): sample at 800 °C).

To better understand the effect of temperature on the sample’s morphological properties at a microscopic scale, a SEM analysis of the studied samples was performed at low and high magnifications. Figure 7 shows the photomicrographs from the analysis. As noticed using the thin-section approach, different minerals can be identified: quartz, plagioclase, and carbonate materials. It can be observed that the primary minerals’ morphology was completely modified by intense metamorphic deformation (Figure 7a–f). This observation agrees with that of the thin section. Comparing the sample at room temperature, 22 °C (Figure 7a,b) with that heated to 600 (Figure 7c,d) and 800 °C (Figure 7e,f), the findings show that at the microscopic scale, no cracks can be noticed for both the samples at 22 and 600 °C, whereas microcracks can be observed on the sample at 800 °C (as indicated by lines on Figure 7f). These show that microcracks were developed on the tested samples macroscopically but had no significant impact on the particles at the microscopic level (Section 3.5.2). It can also be seen that mineral alteration occurred on the sample heated to 800 °C, illustrated by an oval shape in Figure 7f, and can be attributed to the heating of the studied rock samples to 800 °C, which may significantly degrade their mechanical properties.

### 3.4. XRD Analysis

**Figure 8:**
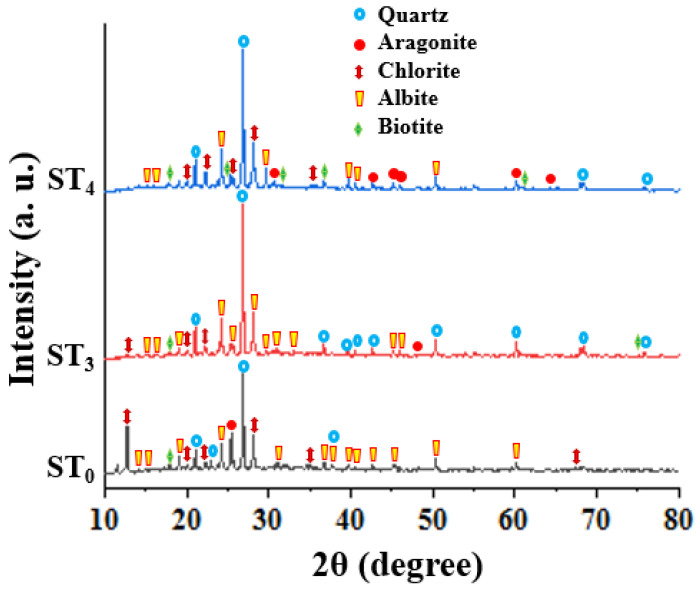
Mineral phases in the studied samples using XRD method (ST_0_–sample at 22 °C; ST_3_–sample heated to 600 °C; ST_3_–sample heated to 800 °C).

To study the mineralogy of the samples and the effect of temperature on the mineral phases in the studied rock samples further, XRD analysis was also employed. The results show that the mineral phases in the studied samples are quartz, aragonite, chlorite, albite, and biotite (Figure 8). These results reveal that the carbonate material that was not identified by both the thin section and SEM analyses is aragonite. The XRD analysis also confirmed that the albite’s plagioclase is identified similar to the results obtained by the thin section and SEM approaches. Meanwhile, chlorite and biotite are also confirmed by the XRD method as identified using the thin-section method. Sericite being suspected to be present by the thin-section method cannot be identified using the XRD method, suggesting that this mineral is not present in the studied samples as any of the employed methods cannot verify it. The results of the quantitative analysis indicate that there are variations in the mineral quantities (weight %) in the three samples, which confirms the anisotropic nature of meta-reworked rhyolitic tuff [51]. This can also be noticed in the spectra as slight differences occur in peaks. Nevertheless, this factor was considered during sample categorization for different target temperatures for quality assurance purposes. It can be seen in Figure 8 that after heating the samples, the peak intensities increased, and a slight phase shift can be noticed. This may lead to alteration in mechanical properties of the studied samples at a macroscopic scale due to the changes in crystallographic directions.

### 3.5. Physical Properties

#### 3.5.1. Density and P-Wave Velocity

**Figure 9:**
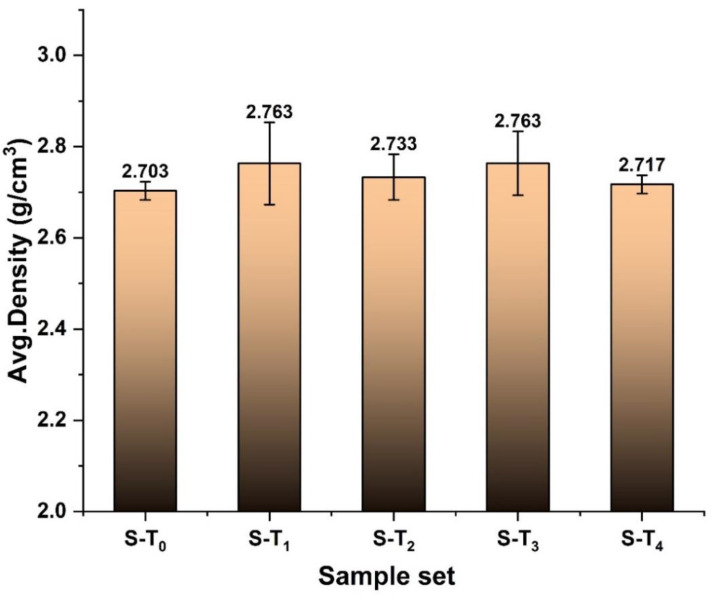
The average density for a different set of specimens tested at different temperatures.

**Figure 10:**
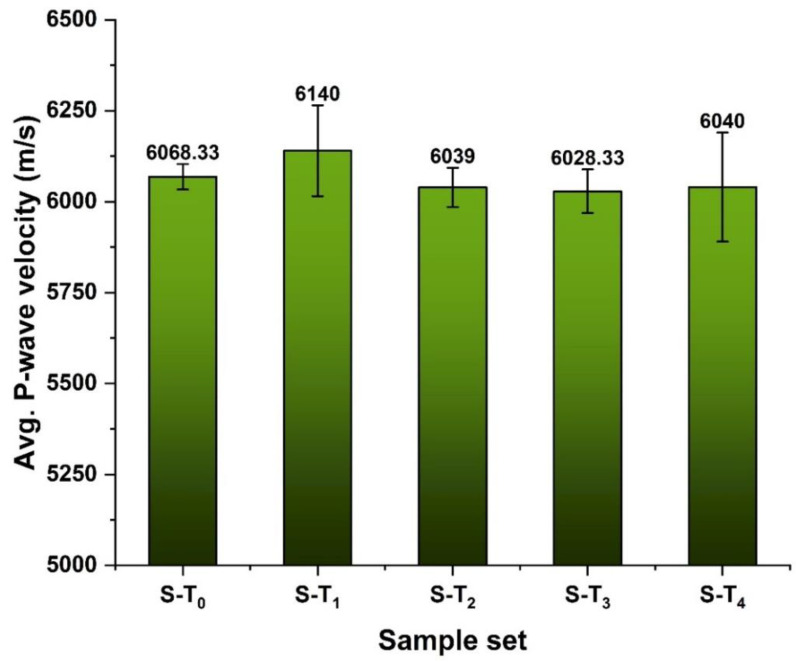
The P-wave velocity of the categorized set of specimens.

**Figure 11:**
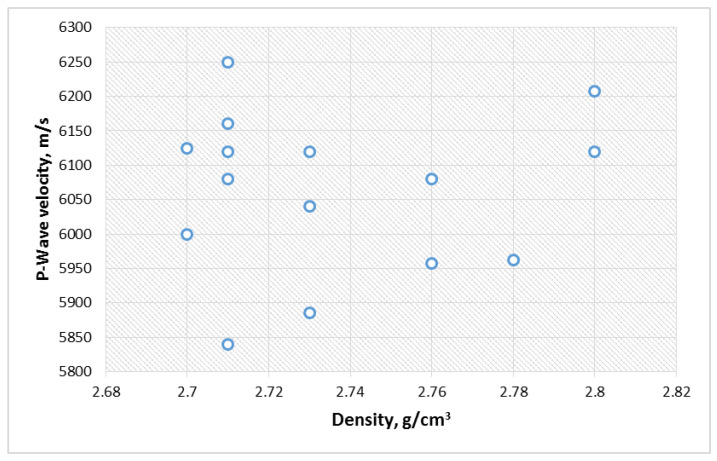
Correlation between P-wave velocity and density of the studied samples.

Table 3 presents the measured dry mass (M), diameter (d), length (L), calculated volume (V), density (D) and the P-wave velocity (V_p_) of the studied samples. Due to the non-homogenous nature of the metamorphic rock, the average density of different specimens slightly varies with the standard variation of 0.024 (Figure 9). The P-wave velocity of the investigated different sets of specimens also varied with a standard variation of 40.65 (Figure 10). Despite the non-homogenous nature of the studied samples, the adopted sample categorization provides close density and P-wave velocity values, which are usually employed in sample selection operations [20]. However, a correlation between the two parameters cannot be established, as presented in Figure 11. This may be related to the variation in the quartz-carbonate vein in the studied samples.

#### 3.5.2. Crack, Color, and Volume of Permeable Pore Space

**Figure 12:**
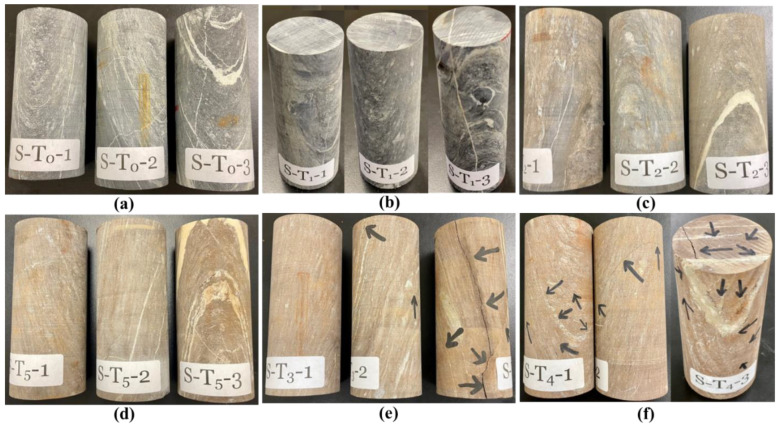
Crack observation of the samples at different temperatures (**a**) 22 °C, (**b**) 200 °C, (**c**) 400 °C, (**d**) 500 °C, (**e**) 600 °C, and (**f**) 800 °C.

**Figure 13:**
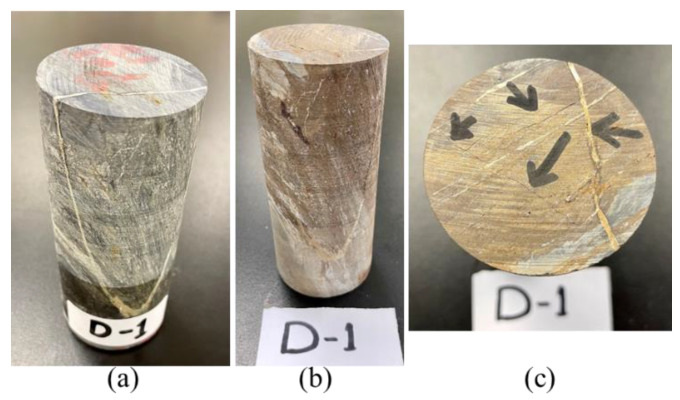
(**a**) A specimen at room temp, (**b**) A specimen after heating to 600 °C, and (**c**) visible cracks after heating to 600 °C.

Figure 12a–f compares samples’ physical appearance (crack and color) at different temperatures. It can be observed that the color of the samples changed from gray (for unheated samples—22 °C; Figure 12a) to brown (for heated samples). As the temperature increases, the samples’ surfaces become deep-brown to reddish-brown, which can be noticed from 400 °C and above (Figure 12c–f). Similar observations have been reported in the literature for limestone [52], microschist [20], granite, and sandstone [23]. It can be seen that microcracks and major-cracks were developed on the specimen (indicated by black arrows) after heating to 600 °C and above (Figure 12e,f). This observation is similar to that of mudstone investigated within the same temperature range as in this study. It was reported that no microcracks were developed on the mudstone samples below 600 °C, after which abundant microcracks were noticed in different directions [53]. Despite the developed cracks, no spalling of particles was noticed on the rhyolitic tuff rock’s surfaces from the samples (Figure 12). The results also indicate that the volume of permeable pore space increased from 0.29 to 1.48% after heating to 600 °C (Table 4). The results can be explained due to the internal structure of the specimens may have been slightly altered, which may lead to crack opening and thus affect their mechanical properties (Figure 13). 

### 3.6. Mechanical Properties

#### 3.6.1. Failure Observations after Uniaxial Loading

**Figure 14:**
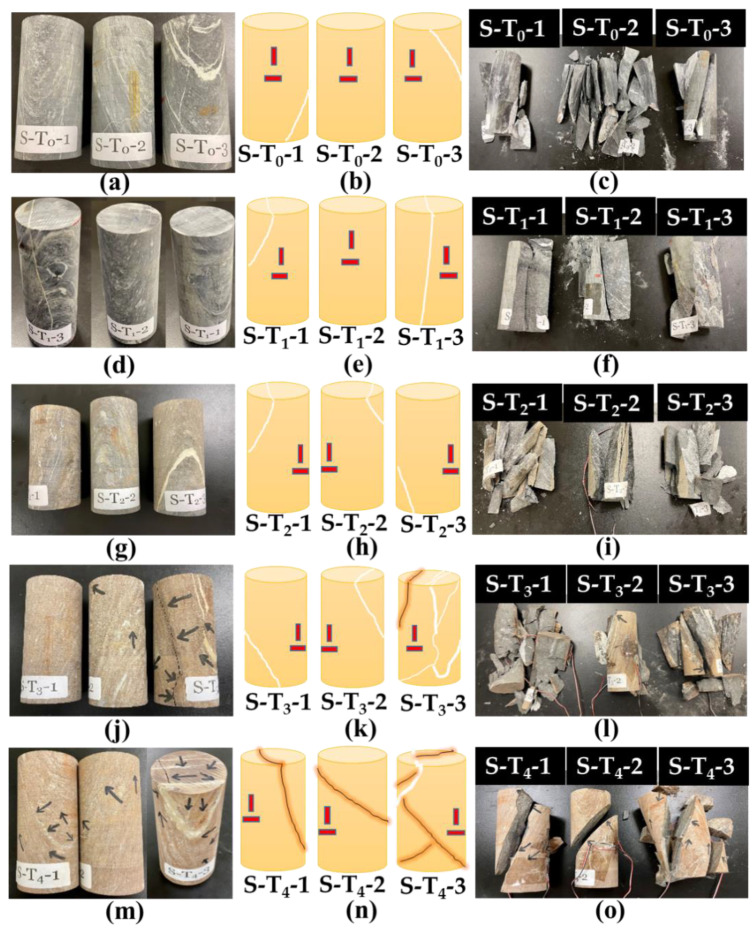
Specimens before and after pressure tests: (**a**,**c**); specimens before and after UCS testing at 22 °C, (**d**,**f**); specimens at 200 °C, (**g**–**i**); specimens at 400 °C, (**j**–**l**); specimens at 600 °C, (**m**–**o**); specimens at 800 °C. (**b**,**e**,**h**,**k**,**n**) represent the schematic specimens with strain gauge (red color), quartz vein (white), crack lines (dark-brown). (**c**,**f**,**i**,**l**,**o**) represent specimens after pressure tests.

Figure 14 presents the specimens before and after heating (Figure 14a,d,g,j,m), schematic diagram of the samples; showing quartz-bedding-planes with positions of pasted strain gauges (Figure 14b,e,h,k,n), and failed specimens after loading (Figure 14c,f,i,l,m). Figure 14a shows the sample S-T_0_-1 to S-T_0_-3; the S-T_0_-1 is an intact specimen free of any visible cracks but with a quartz-bedding plane at the lower part, S-T_0_-2 is also an intact specimen with no quartz-bedding plane, while S-T_0_-3 is an intact specimen with many quartz-bedding planes. After axial compressive loading, S-T_0_-1 (Figure 14c) experienced failure on multiple tension at an angle of 87°. Some cracks were noticed on the foliation plane. For the specimen S-T_0_-2, the applied uniaxial compressive load cannot be sustained by the specimen; leading to its structural burst (Figure 14c; S-T_0_-2). After uniaxial loading on the specimen S-T_0_-3, double shear failure occurred on the bedding plane with a failure angle of 80° (Figure 14c; S-T_0_-3). The specimens S-T_1_-1 to S-T_1_-3 (specimens heated to 200 °C) have different failure behavior after uniaxial compressive loading. The S-T_1_-1 that is free of any visible cracks but with a quartz-bedding plane at the upper part experienced failure at an approximate angle of 90° (Figure 14f; S-T_1_-1). The S-T_1_-2 (intact rock with no quartz-bedding plane) has a single failure with an angle of 70° after pressure load. The S-T_1_-3 (quartz-vein on the specimen) has its failure along quartz-vein with an angle of 80°. As can be seen in the image (Figure 14g), S-T_2_-1 and S-T_2_-2 have small quartz-vein while S-T_2_-3 has quartz-carbonate veins. Multiple fractures occur on S-T_2_-2 while S-T_2_-1 and S-T_2_-3 burst after uniaxial compressive loading (Figure 14i). As presented in Figure 14j, S-T_3_-1 has a single bedding plane with a dip of 75° with a strain gauge vertically placed on the area without bedding (Figure 14k). After uniaxial loading, failure occurs on a non-persistent bedding plane (S-T_3_-1), leading to rockburst (Figure 14l). The specimen S-T_3_-2 (consisting of a quartz-bedding plane at the upper part) developed a major visible crack along the bedding plane and a few minor cracks after heating. Failure of this specimen is on a quartz-bedding plane and persistent bedding plane (Figure 14l; S-T_3_-2). For the third specimen heated to 600 °C (S-T_3_-3; many visible quartz-bedding planes), visible major crack > 1 mm at 80° dip developed due to heating. After loading, failure occurs on the multi-micro-cracks (S-T_3_-3). The specimens S-T_4_-1 and S-T_4_-2 have visible cracks along quartz-bedding-plane with lengths of up to 1 mm from both sides (Figure 14m,n). Failure occurs on a single bedding plane for the two specimens at 75° dip for the former and 53° for the latter (Figure 14o; S-T_4_-1, S-T_4_-2). Major crack > 1 mm at 80° dip developed on the specimen S-T_4_-3 (after heating to 800 °C) mainly along the visible quartz-bedding-plane (Figure 14m,n). Due to loading, failure occurs on this specimen on the bedding plane but with a dip angle 67°, and spallation of particles occurs (Figure 14o; S-T_4_-3). It is important to note that failure mostly occurs along the quartz-bedding plane in all the specimens, whether heated or unheated. Therefore, it is shown that this type of rock may create a serious structural engineering challenge due to its anisotropic nature [51].

#### 3.6.2. Effect of Temperature on UCS and Elastic Modulus of the Studied Samples

**Figure 15:**
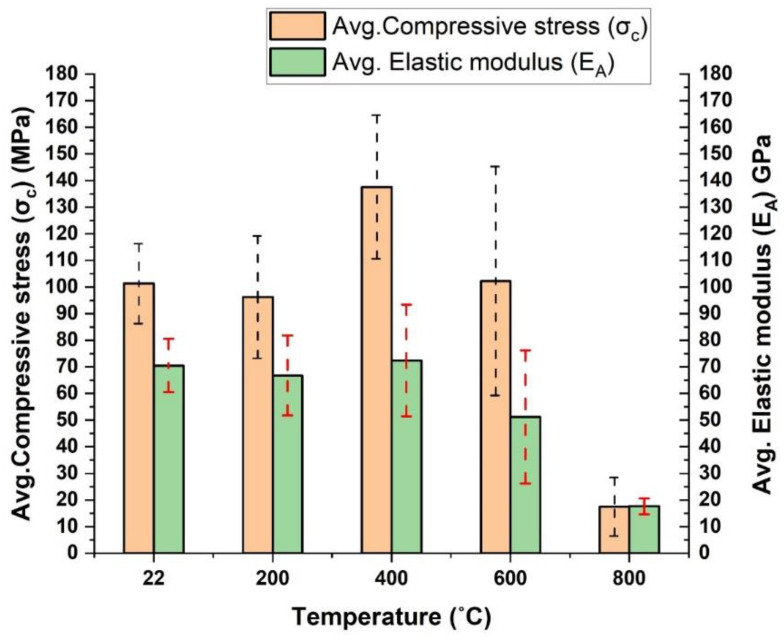
The average compressive stress and elastic modulus for the investigated samples at different temperatures, red and black dotted lines represent uncertainties in stress and elastic modulus.

Table 5 presents the obtained results for the UCS and elastic modulus of the studied meta-reworked rhyolitic tuff samples. The as-received specimens’ average UCS and elastic modulus (22 °C) are 101.29 MPa and 70.51 GPa, respectively. At 200 °C, the average UCS and elastic modulus slightly decreased to 96.20 MPa and 66.78 GPa, equivalent to approximately 5 and 5.3%, respectively. This indicates that increasing temperature (from 22 to 200 °C) reduced the strength of the specimens by nearly 5%. Oppositely, the UCS of the studied specimens increased by 35.8% after heating to 400 °C. Meanwhile, the elastic modulus of the specimens only increased by 2.7% at the same heating conditions. Between 400 to 800 °C, both UCS and elastic modulus follow decreasing trends; as the temperature increases, both mechanical parameters decrease up to 800 °C (Figure 15). At 800 °C, the UCS significantly drops by 82.7%, indicating that the strength of the studied meta-reworked rhyolitic tuff has been degraded. This degradation was due to the expansion of grains that led to substantial microcracks, especially when heated above 400 °C (Figure 14j,m). This indicates that a serious disaster is possible if a fire breaks out in a structure made of meta-reworked rhyolitic tuff and the temperature rises to approximately 600 degrees Celsius or higher. Therefore, the heating-resistant lining is recommended in such structures to avoid unforeseen fire-related structural disasters, especially in an underground structure [37].

**Figure 16:**
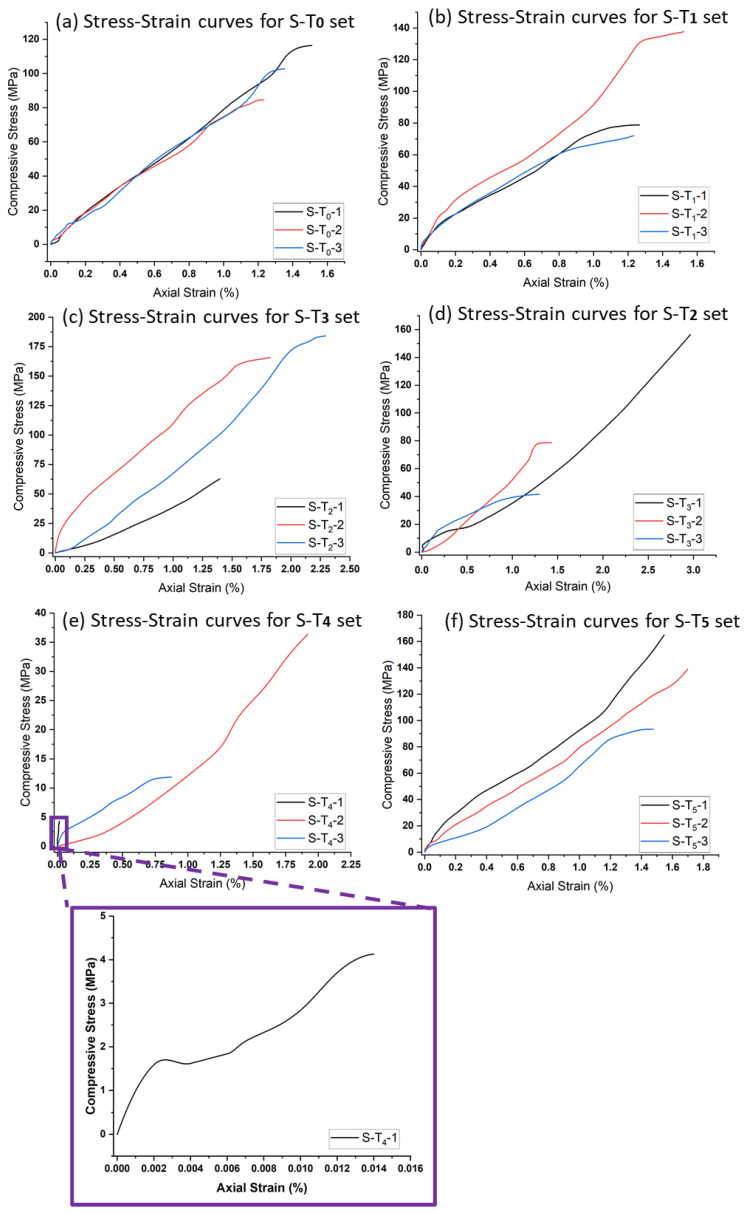
The stress–strain curves of the investigated samples at different temperatures: (**a**) specimens at 22 °C, (**b**) specimens at 200 °C, (**c**) specimens at 400 °C, (**d**) specimens at 600 °C, (**e**) specimens at 800 °C, and (**f**) specimens at 500 °C.

The relationship between σ_c_ and E_A_ is established for all specimens at different target temperatures. The results indicate a linear relationship with convergence behavior between the two parameters for all unheated specimens (Figure 16a). Additionally, there exists a direct relationship between σ_c_ and E_A_ for the specimens heated to 200 °C (Figure 16b), but there was a divergence between the curves, and they differ in their extension, which indicates the beginning of a heating effect on the three specimens that are neither completely the same in mineral composition, nor in their content of quartz-carbonate vein. By examining the stress–strain curve of the specimens heated to 400 °C (Figure 16c), it can be observed that the spacing between the curves increased, which confirms the existence of an effect of heating that caused the difference in the behavior of the three specimens under compression force, more so than the previous set. As for the specimens heated to 600 °C, the relationship between σ_c_ and E_A_ changed (Figure 16d) and completely differed from the specimens heated to 400 °C. As previously mentioned, microcracks were developed on the specimens heated above 400 °C, which may be responsible for the observed changes in the σ_c_ and E_A_ relationship. In addition, quartz phases from α to β usually occur around 573 °C, which may lead to the structural, behavioral change of the specimens noted on their mechanical properties [18]. As the temperature increased to 800 °C, more major and minor cracks were developed (depending on mineral composition and quartz-carbonate veins in the specimens), and the specimens showed different behavior to uniaxial compressive loading. Due to the strong major cracks developed on the specimen after heating, its UCS value is very low and a small strain reading was obtained, as noted on the stress–strain curve (Figure 16e). 

Based on the observed variations in both the UCS and elastic modulus of the samples studied, a set of three samples was selected, which were then heated to 500 °C (the mean temperature between 400 and 600 °C), and both parameters were determined as previously mentioned (Table 5). The findings indicate that at 500 °C, the specimens’ average UCS and elastic modulus are 132.30 MPa and 76.46 GPa, respectively. By looking at all three selected samples at each tested temperature, the results show that the USC of the tested samples initially decreases after heating to 200 °C, increases to 500 °C, has almost the same value as the unheated samples at 600 °C, and then drastically decreases at 800 °C (Figure 15). The specimens show similar behavior for elastic modulus as that of UCS up to 400 °C (Figure 15). Nevertheless, at 500 °C, the elastic modulus of the studied samples slightly increased further before showing decreasing trend at 600 °C and above. A similar complex change in mechanical properties has been reported for sandstones after heating to different temperatures [17]. 

The rejection of a sample with odd value (considering samples with similar quartz-carbonate properties) at any tested temperature. (Table 5), the results indicate that both UCS and elastic modulus decreased after heating to 200 °C. Meanwhile, the apparent increase in rock strength occurs above 200 °C up to 500 °C. At 400 °C, the highest apparent increases of 59.5% and 15.6% were obtained for the UCS and elastic modulus, respectively. At 600 and 800 °C, the strength of the selected investigated samples dropped by 45% and 78%, respectively, equivalent to 41.3% and 75.9% reductions in elastic modulus. The hitherto results suggest that the threshold temperature of the studied rhyolitic tuff is within 500 to 600 °C. This is higher than that of the granite (having threshold temperature around 400 °C) [34,35,36], carbonate rocks (threshold temperature within 300 to 400 °C), and sandstones (threshold temperature–300 to 500 °C) but lower than that of a shale (threshold temperature–600 to 700 °C) [38]. However, the obtained threshold temperature in this study has the same range as reported for the mudstones (500 to 600 °C) [38]. Nevertheless, the threshold temperature and other engineering properties of heated rocks depend on their geological origin, mineral compositions, stress conditions, and microstructural defects [39]. By comparing the results of the whole set of samples with those of selected samples (with similar quartz-carbonate property), it can be deduced that the bedding in the samples is responsible for a further 4.7% deterioration in the strength of the rocks at 800 °C.

**Figure 17:**
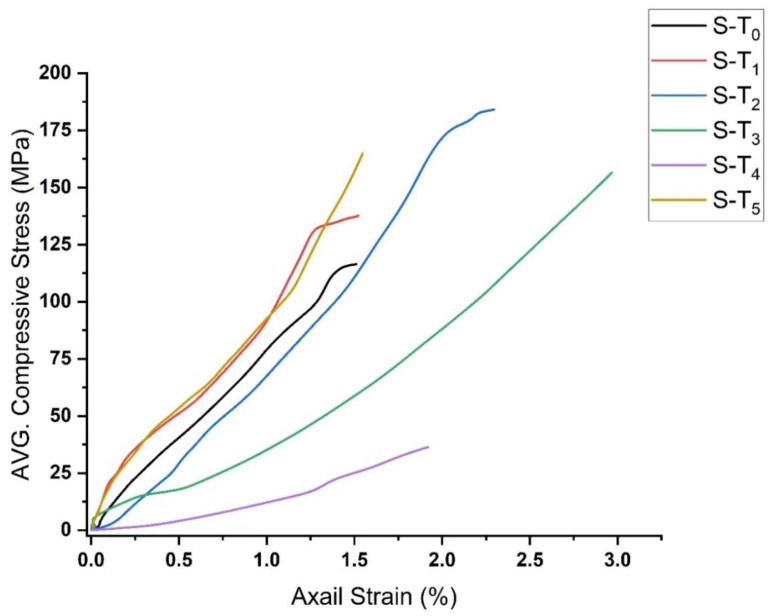
Comparison of the stress–strain curves of the investigated specimens at different temperatures (S-T_0_; specimens at 22 °C, S-T_1_; specimens at 200 °C, S-T_2_; specimens at 400 °C, S-T_3_; specimens at 600 °C, S-T_4_; specimens at 800 °C, S-T_5_; specimens at 500 °C.

Figure 17 compares the stress–strain curves of the investigated samples at different temperatures. It can be noticed that the specimens heated to 200 and 500 °C show a similar stress–strain relationship up to around 80 MPa with 1.0% change in axial strain, above which divergence behavior can be observed. Apart from these temperatures, the specimens show a pronounced stress–strain relationship with a tendency in the direction of the axial strain axis. The specimens heated to 400 °C showing the highest average UCS value and the specimens heated to 800 °C showing the lowest average UCS value, which can be attributed to the elongation of microcracks and the propagation of new visible cracks due to the expansion of the grains [20].

## 4. Conclusions

◦ The current work studied the rhyolitic tuff rocks; thus, the physical, mechanical, and microstructural properties were investigated at different target temperatures. The results showed that heating the meta-reworked rhyolitic tuff rocks causes a change in the physical appearance of the samples tested; hence, the samples became brownish (from gray) after heating to 600 °C. Small and large cracks observed after heating to 600 °C and higher.
◦ However, heating meta-reworked rhyolitic tuff rocks have two opposing impacts on their mechanical characteristics. Due to the growth of internal fractures towards the surface, they constitute weakness planes that induce early failure in the examined samples.
◦ In the rhyolitic tuffs studied, the mechanical properties (UCS and Young’s modulus) decrease drastically beyond 500–600 °C. After heating to 800 °C, the UCS and elastic modulus of selected, consistent, and nearly homogeneous rhyolitic tuff samples dropped by 78.0 and 75.9%, respectively. At this temperature, the UCS of all rhyolitic tuff samples dropped by 82.7% (including samples with bedding planes).
◦ The SEM images of the heated samples show structural particle displacements and microcracks that support the visible surface fractures (at the macroscopic scale). It is important to consider temperature effects when constructing structures from meta-reworked rhyolitic tuffs to minimize structural collapse. Thus, to prevent structural damage at 800 °C, an additional 4.7% strengthening is required.

## Figures and Tables

**Table 1:** Effect of temperature on mechanical rock properties.

Rock Sample	Temp. Range (°C)	Heating Rate (°C/Min)	Studied Property	Results	Reference
Granite	25–1000	20	Elastic modulus	Static elastic modulus fluctuated below 400 °C, sharply decreased between 400 and 600 °C, and decreased up to 83.3% at 1000 °C	[1]
			UCS	Decreased up to 75.8% at 1000 °C	
Granite	25–800	2	Elastic modulus	Elastic modulus decreased at all studied temperatures when compared with the samples at room temperature.	[40]
Sandstone	20–300	30	UCS, elastic modulus, and tensile strength	All studied properties linearly increased at low temperature up to between 200 to 250 °C and thereafter decreased.	[29]
Sandstone	20–800	30	UCS, strain, elastic modulus	UCS decreased between 20–200 °C, increased between 200 and 500 °C, and then decreased up to 800 °C. Strain decreased between 20 and 200 °C and then increased up to 800 °C. Elastic modulus fluctuated throughout the studied temperatures.	[41]
Marble	25–600	10	UCS, elastic modulus	Both parameters decreased with temperature.	[42]
Granodiorite	200–800	5	Tensile strength	Tensile strength decreased at all studied temperatures when compared with the samples at room temperature.	[28]
Limestone	25–900	5	Elastic modulus	Elastic modulus decreased at all studied temperatures when compared with the samples at room temperature.	[43]

**Table 2:** Weight % of elements in the studied samples ST_0_–ST_4._

Sample Status	Elements										
	C	O	Na	Mg	Al	Si	K	Ca	Ti	Fe	Cu
ST_0_	5.93	53.48	5.48	0.70	8.23	22.09	0.65	0.54	-	2.90	-
ST3	2.33	55.20	4.14	0.73	7.13	24.93	0.46	0.83	0.61	3.63	-
ST4	4.87	49.35	0.91	1.74	9.41	21.70	2.82	0.60	-	7.56	1.04

**Table 3:** The specimens’ density and P-wave velocity (Vp) at room temperature (22 °C).

Sample ID	Density Calculation					P-Wave Velocity
	M (g)	Avg. d (mm)	Avg. L (mm)	V (cm^3^)	D (g/cm^3^)	V_p_ (m/s)
S-T_0_-1	1242.73	63.66	144.82	461.06	2.70	6125
S-T_0_-2	1290.53	63.76	149.40	477.21	2.70	6000
S-T_0_-3	1309.83	63.70	151.48	482.93	2.71	6080
S-T_1_-1	1335.23	63.70	149.37	476.13	2.80	6208
S-T_1_-2	1277.03	63.59	148.50	471.81	2.71	6250
S-T_1_-3	1380.43	63.72	155.67	496.52	2.78	5962
S-T_2_-1	1201.03	63.68	136.70	435.47	2.76	5957
S-T_2_-2	1319.13	63.69	152.72	486.65	2.71	6120
S-T_2_-3	1311.33	63.66	150.63	479.56	2.73	6040
S-T_3_-1	1362.83	63.64	152.97	486.70	2.80	6120
S-T_3_-2	1338.63	63.74	153.56	490.19	2.73	5885
S-T_3_-3	1320.63	63.66	150.34	478.71	2.76	6080
S-T_4_-1	1309.13	63.75	151.54	483.88	2.71	6160
S-T_4_-2	1287.83	63.72	149.15	475.80	2.71	5840
S-T_4_-3	1309.23	63.68	150.80	480.40	2.73	6120

**Table 4:** Volume of permeable pore space (voids, %).

Temperature (°C)	Sample ID	Dry Bulk Density (g/cm^3^)	Bulk Density after Immersion (g/cm^3^)	Bulk Density after Immersion and Boiling (g/cm^3^)	Apparent Density	Volume of Permeable Pore Space (Voids), %
22 (room)	D-1	2.80	2.81	2.81	2.81	0.29
600	D-1	2.73	2.74	2.74	2.77	1.48

**Table 5:** Results of UCS and elastic modulus of the specimens.

Sample ID	Temperature (°C)	Compressive Strength, σ_c_ (MPa)	Elastic Modulus, E_A_ (GPa)
S-T_0_-1	22	116.45	75.43
S-T_0_-2		84.58	64.71
S-T_0_-3		102.83	71.40
S-T_1_-1	200	78.78	63.32
S-T_1_-2		137.74	77.25
S-T_1_-3		72.07	59.76
S-T_2_-1	400	62.88	47.46
S-T_2_-2		165.75	82.66
S-T_2_-3		184.10	87.13
S-T_3_-1	600	186.06	67.36
S-T_3_-2		78.58	54.75
S-T_3_-3		42.12	31.52
S-T_4_-1	800	4.30	0.00
S-T_4_-2		36.39	19.76
S-T_4_-3		11.89	15.56
S-T_5_-1	500	164.91	82.05
S-T_5_-2		138.63	78.16
S-T_5_-3		93.35	69.18

## Data Availability

Not applicable.
